# Account of Graefe's Ligature of the Arteria Innominata

**Published:** 1823-06

**Authors:** 

**Affiliations:** Berlin.


Art. VI.—*Account of* Graefe's *Ligature of the Arteria Innominata.* Communicated in a Letter to Dr. Macleod, from Professor Wagner, of Berlin.

Professor Graefe, of Berlin, tied the arteria innominata on the 5th of March, 1822, in a sailor, about thirty years of age, who was affected with an aneurism in the right subclavian artery. During the operation, the patient was put upon his back on a table, in such a manner that his head was hanging down on one side of the table, and the arteria innominata drawn up a little above the manubrium of the sternum. The Professor then made a longitudinal incision, near the anterior edge of the sterno-mastoid muscle, down to the sternum, exposed the carotid artery, and followed its course till where it unites with the subclavian artery to form the trunk of the arteria innominata. This was effected with very little difficulty, and the arteria innominata became distinctly exposed to sight. By means of a needle, curved in a particular manner for that purpose, a ligature was then passed round the arteria innominata, and both ends of it tied together.

No alarming symptom ensued, and the ligature, which was considerably broad, came away about a fortnight after the operation, carrying out just that portion of the artery where the innominata is divided into the carotid and subclavian artery. Some time afterwards, however, a hemorrhage suddenly arose, which, though considerable, was soon stopped by the application of cold water and pressure. The patient then complained of pains in the tumor formed by the aneurism, in which fluctuation was distinctly to be felt; and this determined Dr. Gräefe to make an incision into the aneurismal sac. A considerable quantity of pus and grumous blood was thus evacuated; and matter continued to be discharged daily from this opening, whilst the other was filled with granulations, and went on exceedingly well. The patient, however, got into a state of fever, began to spit blood, and to throw up matter; and so he died on the sixty-eighth day after the operation had been performed.

On dissection, the lungs were found in a diseased state; and in the arteria innomina[illegible]a a clot had been formed, extending from its origin to where [illegible]he ligature had been applied. By an injection made into the ao[illegible]ta, the arteries of the right arm and right side of the head were entirely filled; the circulation in these parts having been fully re-established by anastomosing branches.

